# A case report of blood pressure fluctuation, inverted Takotsubo cardiomyopathy, and pulmonary embolism: searching for a link

**DOI:** 10.1093/ehjcr/ytaf574

**Published:** 2025-11-01

**Authors:** Alberto Mazza, Claudio Picariello, Maurizio De Luca, Elisa Canova, Marco Zuin

**Affiliations:** Internal Medicine and ESH Excellence Hypertension Center, Santa Maria degli Angeli General Hospital, Piazzale degli Etruschi, 6, Adria (Rovigo) 45011, Italy; Department of Cardiology, Santa Maria della Misericordia General Hospital, 45100 Rovigo, Italy; Department of Surgery, Santa Maria della Misericordia General Hospital, 45100 Rovigo, Italy; Department of Pathology, Santa Maria della Misericordia General Hospital, 45100 Rovigo, Italy; Department of Translational Medicine, University of Ferrara, 44124 Ferrara, Italy; Department of Cardio-Thoraco-Vascular Sciences and Public Health, University of Padova, 35128 Padova, Italy

**Keywords:** Hypotension, Hypertension, Pulmonary embolism, Takotsubo, Case report

## Abstract

**Background:**

Alternating cycles of hypertension, sustained hypotension, and heart rate (HR) variability, manifesting as an inverted pattern of Takotsubo cardiomyopathy (ITC) and pulmonary embolism (PE) due to an underlying previously undiagnosed pheochromocytoma (Pheo), is a rare and complex clinical presentation. To date, only two previous reports have described this triad. Despite the absence of established guidelines for managing blood pressure (BP) and HR fluctuations in patients with Pheo, understanding the interplay between these conditions is crucial for improving patient outcomes and optimizing treatment strategies. This case report highlights the diagnostic challenges and the need for further investigation into effective management approaches in such patients.

**Case summary:**

We present a case of a 68-year-old man hospitalized for acute chest pain and BP and HR fluctuations. The clinical work-up revealed the coexistence of ITC, PE, and Pheo. The patient was successfully treated with continuous volume expansion and selective α_1_-adrenergic blockade, avoiding vasodilators and renin-angiotensin-aldosterone inhibitors. Perioperative administration of oral doxazosin and bisoprolol allowed a successful laparoscopic right adrenalectomy after 3-month anticoagulation.

**Discussion:**

Despite the lack of established recommendations for managing BP and HR fluctuations in patients with Pheo, the coexistence of PE further complicates the clinical management of this condition, suddenly modifying the haemodynamic status and increasing the risk of major bleeding events from the neoplastic mass. Patients presenting with ITC, PE, and Pheo require a multidisciplinary approach involving a cardiologist, hypertension specialist, and surgeon to resolve this intriguing clinical condition.

Learning points

- Inverted Takotsubo cardiomyopathy, pulmonary embolism, and pheochromocytoma are a rare triad.
- In these patients, haemodynamic instability demands personalized management.

Inverted Takotsubo cardiomyopathy (ITC) is characterized by transient left ventricular (LV) wall motion abnormalities predominantly affecting the basal and mid-ventricular segments, with preserved apical contractility, in the absence of significant coronary artery stenosis.^1^ While the real pathophysiological mechanisms remain unclear, catecholamine excess and increased sympathetic activity are thought to play a key role in triggering ITC. Moreover, the link between acute pulmonary embolism (PE) and Takotsubo cardiomyopathy (TTC) has not been fully elucidated. Differential diagnosis between Takotsubo and acute PE remains a challenge since both clinical presentations often mimic an acute coronary syndrome.

We present the case of a middle-aged man who experienced ITC with concomitant acute PE and an unknown adrenal pheochromocytoma (Pheo) showing recurrent, prolonged, and sustained hypotensive episodes and heart rate (HR) variability.

## Summary figure

**Figure:**
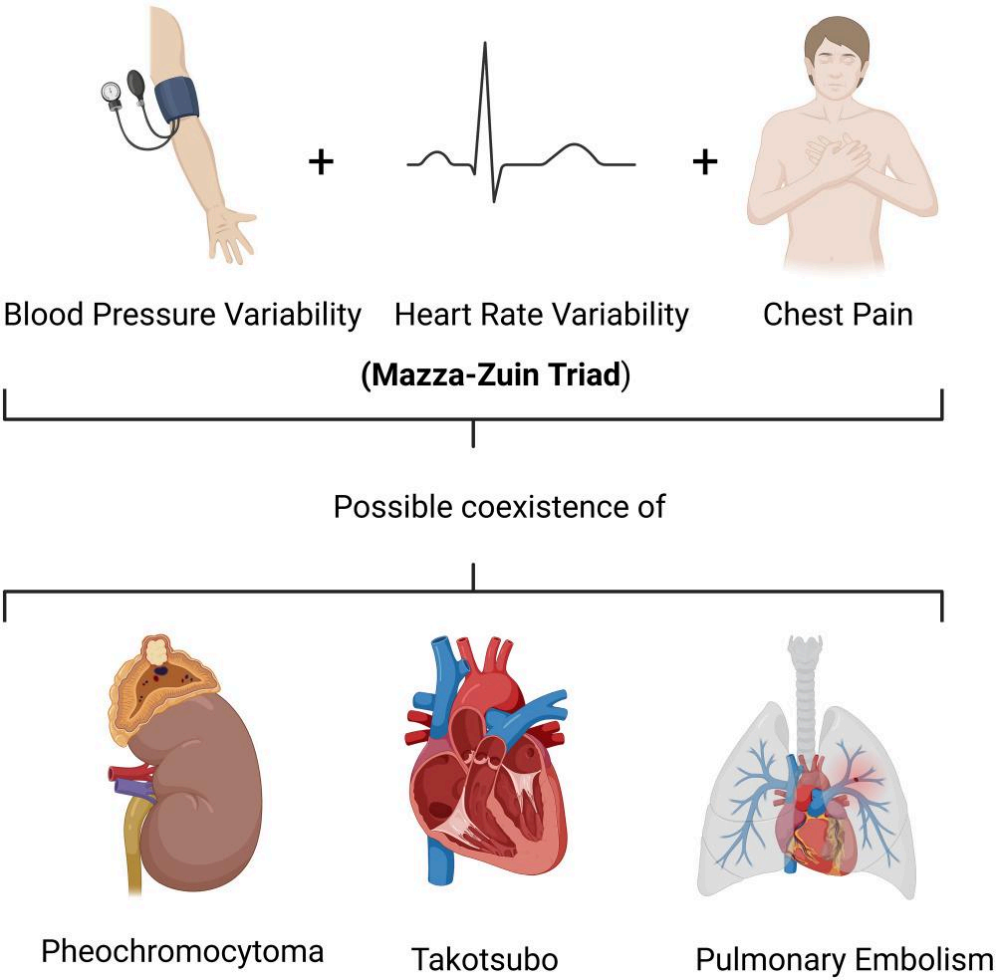


## Case presentation

A 68-year-old man was admitted to intensive cardiac care unit (ICCU) for typical chest pain, worsening dyspnoea, 37.7°C temperature, and mildly elevated blood pressure (BP) (158/94 mmHg) and HR (102 b.p.m.) values. Previous medical history included arterial hypertension treated with olmesartan + amlodipine 20 + 5 mg/day, Stage II-B peripheral artery disease managed with 100 mg/day acetylsalicylic acid, left hernioplasty, and a previous endoscopically treated bladder cancer.

**Figure 1:**
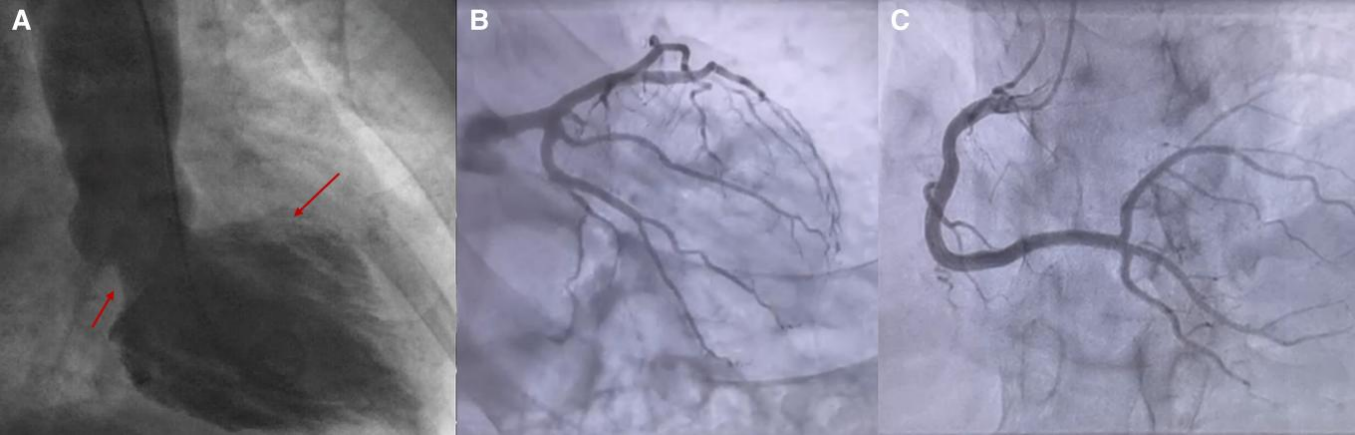
Cardiac ventriculography reveals a pattern characteristic of inverted (basal) Takotsubo cardiomyopathy, with hyperkinesis of the apical segments and hypokinesis of the basal left ventricular walls (*A*, arrows). Coronary angiography reveals no obstructive coronary artery disease (*B* and *C*).

Initial blood tests revealed elevated ultrasensitive troponin T (194 ng/L, normal value < 15 ng/L) and ST-segment depression in V_3_–V_6_ leads. Transthoracic echocardiogram (TTE) showed left ventricular (LV) hypertrophy with preserved LV ejection fraction (LVEF) (58%) and basal/mid-ventricular segmental hypokinesia with preserved apical segmental function. Given persistently serial troponin T measurements (peaking of 456 ng/L after 4 h), left ventriculography was performed and confirmed ITC cardiomyopathy with normal apical motion and akinesias in the other LV parts (*Figure 1A*), while coronary angiography reveals no obstructive coronary artery disease (*Figure 1B* and *C*).

**Figure 2:**
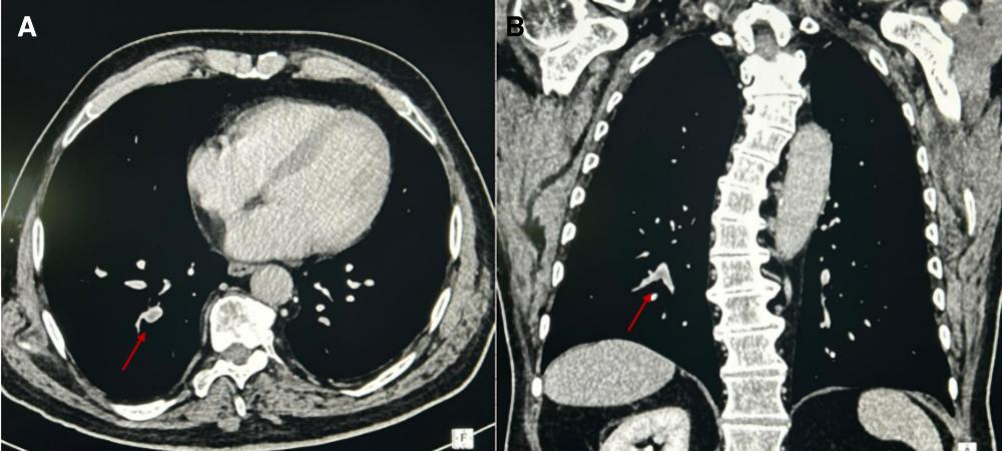
Computed tomography pulmonary angiography showing multiple filling defects in the segmental branches of the pulmonary arteries, consistent with pulmonary embolism (*A* and *B*, arrows; coronal and sagittal planes, respectively). CTPA, computed tomography pulmonary angiography.

The following day, D-dimer serum levels increased until 3.72 mg/L (normal value < 0.5 mg/L). Thoracic computed tomography angiography (CTPA) revealed segmental PE in the right lower lobe segmental branch (*Figure 2 A* and *B*). Transthoracic echocardiogram confirmed preserved LVEF and a non-dilated normokinetic right ventricle (tricuspid annular plane systolic excursion 20 mm). The patient was classified as intermediate–low-risk PE and received oral anticoagulation with rivaroxaban 15 mg twice daily. Lower extremity colour-Doppler ultrasound excluded deep vein thrombosis.

Moreover, the CTPA scan, extended to the lower thorax, revealed a 5 × 3 cm right adrenal mass. Further TTE on the same day showed recovery of contractile function with normal biventricular function.

**Figure 3:**
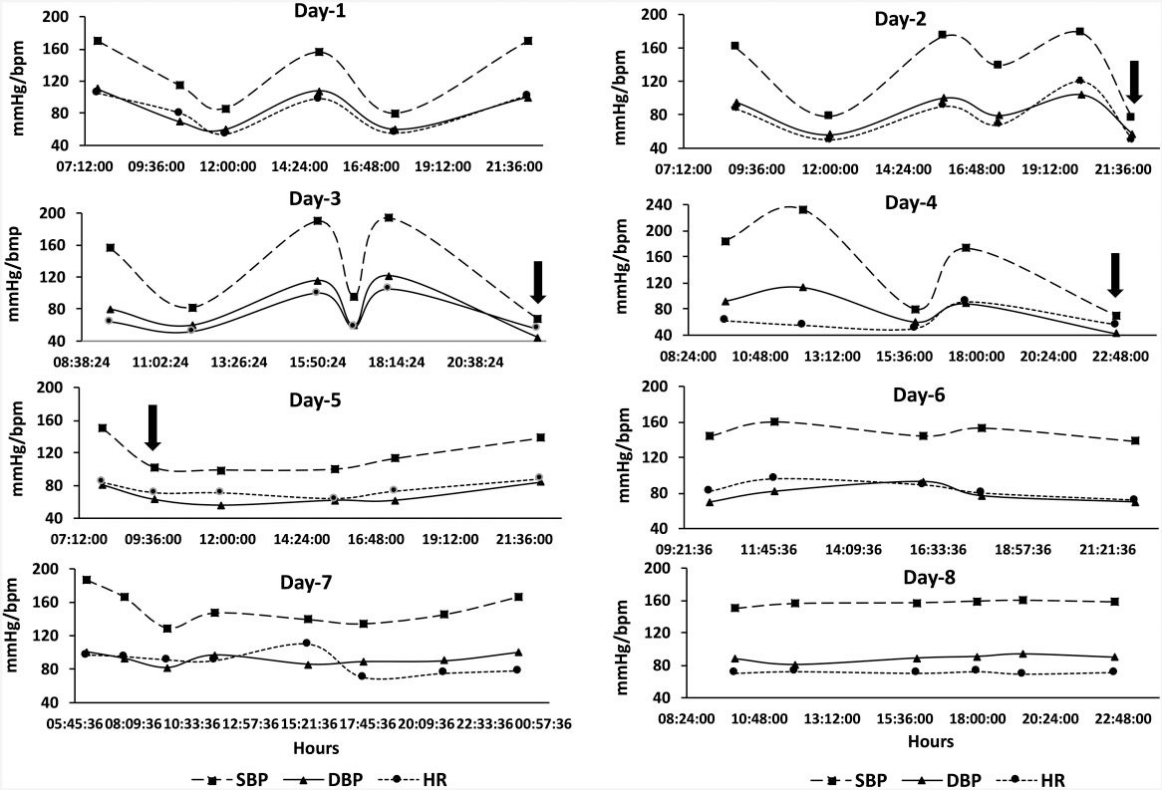
Intra-arterial monitoring of systolic blood pressure (mmHg), diastolic blood pressure (mmHg), and heart rate (b.p.m.) from Days 1–8. Significant reductions in systolic blood pressure and heart rate were noted on Days 2, 3, and 4 (black arrows). After initiation of alpha-blockade and intravenous fluid therapy, fluctuations in systolic blood pressure, diastolic blood pressure, and heart rate decreased markedly by Day 5. On Days 6 and 7, haemodynamic variability persisted but became less frequent and more irregular. Haemodynamic stabilization was achieved by Day 8, following the introduction of oral doxazosin. SBP, systolic blood pressure; DBP, diastolic blood pressure; HR, heart rate.

During the second day of hospitalization in ICCU, the patient experienced symptomatic hypertensive crisis with BP peaking to 240/130 mmHg, chest pain, palpitation, headache, and diaphoresis. Intra-arterial BP and HR monitoring revealed marked fluctuations with a roughly 2-h cycle, where systolic BP (SBP) and HR changed reciprocally (*Figure 3*). Nitro-glycerine infusion improved symptoms, lowering BP to 120/80 mmHg and HR to 50 b.p.m. To prevent further BP increases, furosemide 25 mg and spironolactone 37.5 mg daily were added to the pre-existing olmesartan + amlodipine regimen. However, over the subsequent 4 days, intra-arterial SBP and HR continued to fluctuate ranging from 68 to 233 mmHg and 50 to 120 b.p.m., respectively (*Figure 3*, Days 3 and 4).

Hypertensive crises were managed with intravenous labetalol (100 mg), followed by continuous urapidil infusion at 12.5 mg/h and co-infusion with 3000 mL/day normal saline. This approach, combined with discontinuation of nitroglycerin, calcium antagonists, and drugs inhibiting the renin-angiotensin-aldosterone system (RAAS; i.e. sartan, spironolactone, and furosemide), resulted in the resolution of BP and HR fluctuation cycles (*Figure 3*, Days 5–8). The patient remained in sinus rhythm throughout the entire hospitalization. More precisely, multiple electrocardiograms consistently showed sinus rhythm with incomplete right bundle branch block + left axis deviation, interventricular conduction delay, and non-specific repolarization abnormalities in the right precordial leads (see Supplementary material online, *Figure S1*).

**Figure 4:**
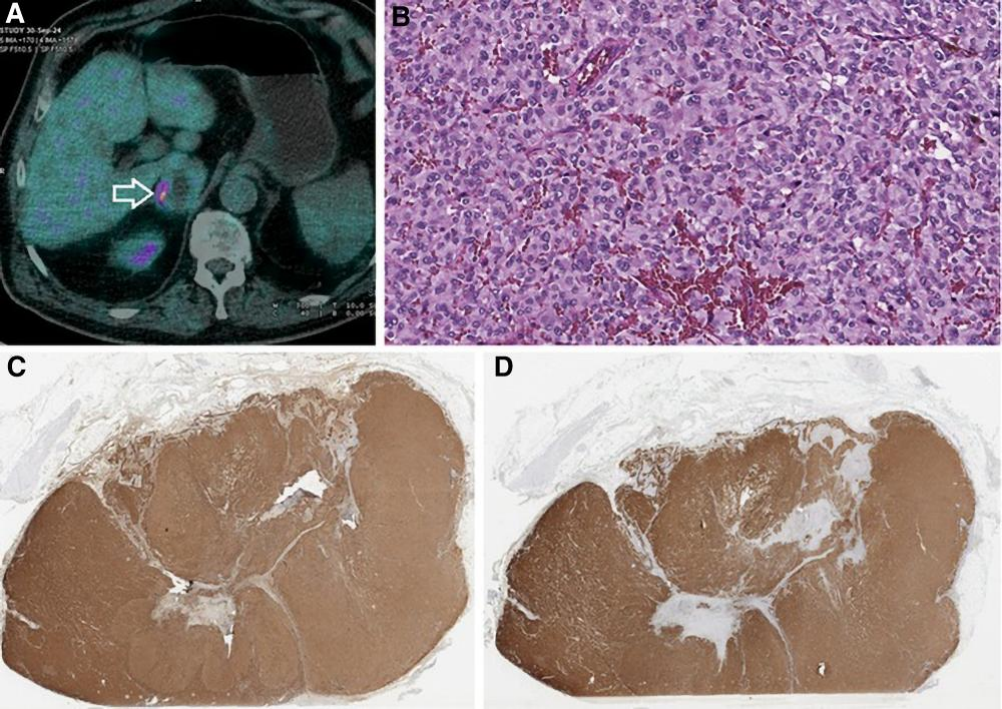
(*A*) F-18 fluoro-dihydroxyphenylalanine (positron emission tomography/computed tomography scan revealing intense radiotracer uptake in the right adrenal gland (arrow), consistent with pheochromocytoma. (*B*) Histopathological examination of the adrenal mass showing a trabecular (Zellballen) pattern composed of large polygonal neoplastic cells with round to oval nuclei, prominent nucleoli, and abundant cytoplasm (H&E, ×20). (*C* and *D*) Immunohistochemistry showing diffuse cytoplasmic positivity for chromogranin A and synaptophysin, respectively. ¹⁸F-FDOPA, F-18 fluoro-dihydroxyphenylalanine; PET/CT, positron emission tomography/computed tomography.

Meanwhile, F-18 fluoro-dihydroxyphenylalanine (^18^F-FDOPA) positron emission tomography with complementary computed tomography (PET/CT) revealed abnormal tracer uptake (Suv Max of 14.23) in the right adrenal gland (*Figure 4A*). Blood tests showed elevated chromogranin A (258.6 ng/L, normal value < 108 ng/mL), cortisol (27 μg/dL, normal value < 12 μg/dL), glycosylated haemoglobin (45 mmol/mol), and 24-h urinary metanephrines (2947 μg/L/24 h, normal value < 260 μg/L/24 h). Other blood parameters, including thrombophilia panel, serum creatinine, potassium, N-terminal pro-B-type natriuretic peptide, thyroid-stimulating hormone, and 24-h urinary cortisol levels as well as the Nugent test were normal.

At discharge, BP and HR were normalized (138/84 mmHg and 70 b.p.m.) with oral doxazosin 8 mg/day oral doxazosin and 1.25 mg/daily oral bisoprolol.

The patient underwent laparoscopic right adrenalectomy after 3-month full oral anticoagulant therapy. Histological examination revealed the typical Zellballen pattern with trabecular arrangement of large and polygonal neoplastic cells, round oval nuclei, prominent nucleoli, and abundant cytoplasm (*Figure 4B*). Moreover, chromogranin (*Figure 4C*), synaptophysin (*Figure 4,D*), and S-100 immunohistochemical staining showed a diffusely positive cytoplasmic granular pattern on neoplastic cells (Ki-67 1–2%). The neoplasm was negative for CK-7, positive for synaptophysin and CD-56, and negative for CK-7 and CK-20. Additionally, immunohistochemistry staining was intense and weak for succinate dehydrogenase subunit-A (SDHA) and succinate dehydrogenase subunit-B gene mutations.

These findings confirmed the final diagnosis of moderately differentiated Pheo, having a Grading System for Adrenal Pheochromocytoma and Paraganglioma (GAPP) score of 5.

Following Pheo resection, antihypertensive therapy with 4 mg doxazosin 4 mg once a day in the evening was continued, and a fixed-dose combination of perindopril + amlodipine (5 + 5 mg once a day in the morning) was initiated, after complete recovery of LVEF.

Rivaroxaban therapy was extended to a total duration of 6 months as continuation of anticoagulation for prevention of recurrence of PE. During the follow-up period, the patient remained asymptomatic, with normalization of BP, and reported satisfaction for her clinical improvement. A timeline summarizing the key clinical events, diagnostic findings, and therapeutic interventions was provided (*Table 1*).

**Table 1:** Clinical timeline summary

Day	Clinical event/finding	Investigations	Treatment/intervention
Day 0	Admission for chest pain, dyspnoea, fever (37.7°C), BP 158/94 mmHg, HR 102 b.p.m.	ECG: ST depression (inferior leads, V3–V6); TTE: basal and mid-ventricular hypokinesia; troponin T: 194 → 456 ng/L	Continued olmesartan/amlodipine; ASA maintained
Day 1	Suspected inverted Takotsubo cardiomyopathy	Coronary angiography: no stenosis; LVG: basal/mid-akinesia, apical sparing	Antiplatelet therapy continued
Day 2	D-dimer increased to 3.72 mg/L	CTPA: segmental PE; Echo: preserved biventricular function	Rivaroxaban 15 mg b.i.d. started
Day 2 (cont.)	Adrenal mass incidentally detected on extended CT	CT: right adrenal mass (5 × 3 cm); Echo: normalization of LV function	–
Day 3	Hypertensive crisis: BP 240/130 mmHg, chest pain, diaphoresis, cyclic BP/HR variation	Intra-arterial monitoring: 2-h BP–HR oscillations	i.v. nitroglycerin → furosemide + spironolactone added
Days 4–5	Persistent BP/HR fluctuations (68–233 mmHg; 50–120 b.p.m.)	—	Initiated i.v. labetalol + continuous urapidil + 3L i.v. saline/day; discontinued nitroglycerin, RAASi, and CCBs
Days 5–8	Stabilization of haemodynamics	BP/HR normalized; patient remained in sinus rhythm	Continued urapidil infusion, preparation for alpha-blockade
Day 9	Etiological work-up of adrenal mass	PET/CT: intense ^18^F-FDOPA uptake; labs: ↑ chromogranin A, urinary metanephrines	Diagnosis of pheochromocytoma confirmed
Discharge (Day 10)	Clinical stability achieved	–	Oral doxazosin 8 mg/day + bisoprolol 1.25 mg/day; rivaroxaban continued
3 months post-discharge	Definitive management	Laparoscopic right adrenalectomy	Histology: moderately differentiated pheochromocytoma

ASA, acetylsalicylic acid; b.i.d., bis in die; BP, blood pressure; CCB, calcium channel blocker; CT, computed tomography; CTPA, computed tomography pulmonary angiography; HR, heart rate; i.v., intravenous; PET/CT, positron emission tomography/computed tomography; RAASi, renin-angiotensin-aldosterone system inhibitors; TTE, transthoracic echocardiography; ^18^F-FDOPA, F-18 fluoro-dihydroxyphenylalanine.

## Discussion

In Pheo, severe hypotension accompanied by marked BP fluctuations is rare, making BP stabilization challenging, especially before Pheo suspicion and diagnosis. While hypertension management in Pheo is well-established, hypotension management lacks consensus and relies on anecdotal reports. Post-resection hypotension often needs continuous vasopressor infusion despite fluid replacement, with norepinephrine or vasopressin used paradoxically before surgery in some cases.^2,3^ Upon ICCU admission, the patient’s antihypertensive regimen paradoxically caused severe hypotension, highlighting the need to avoid vasodilatory agents and renin-angiotensin-aldosterone system inhibitors. Conversely, β-blockade should only be initiated after adequate α-adrenergic blockade.^4^ The patient initially presented with mildly elevated BP and HR and was managed with her chronic antihypertensive regimen in addition to loop- and potassium-sparing diuretics during early hospitalization. However, after the onset of paroxysmal hypertensive crises with symptomatic adrenergic surges, continuous intra-arterial monitoring revealed cyclic fluctuations in BP and HR, suggestive of catecholamine-driven haemodynamic instability. In this context, the use of RAAS inhibitors and vasodilators, which could potentiate hypotensive episodes or paradoxically stimulate compensatory catecholamine release, became potentially harmful. These agents were therefore discontinued on Day 4 of hospitalization. Additionally, α-adrenergic blockade with urapidil infusion was initiated as the first-line therapy to counteract the effects of catecholamine excess and restore haemodynamic stability. Urapidil was preferred due to its dual α1-blocking and central sympatholytic properties. Following stabilization of BP variability and resolution of the fluctuation cycles, oral α-blockade with doxazosin was introduced. Low-dose β-blockade was subsequently added, only after adequate α-blockade was achieved, in accordance with established principles of Pheo management. This sequence was critical to avoid unopposed β-adrenergic vasoconstriction, which could precipitate hypertensive crisis or pulmonary oedema.

Myocarditis was considered in the differential diagnosis, but was deemed unlikely given the absence of viral prodromes, normal inflammatory markers, and the rapid recovery of biventricular function without immunosuppressive therapy. Ischaemic cardiomyopathy was also excluded based on urgent coronary angiography, which demonstrated normal epicardial coronary arteries, and the absence of regional wall motion abnormalities in a coronary distribution. The characteristic basal and mid-ventricular akinesia with apical sparing, observed on ventriculography and echocardiography, was diagnostic for ITC, especially in the context of emotional and physical stressors, including catecholamine surges.

Beyond hypertensive emergencies, this case underscores potential thromboembolic complications in Pheo.^5^ An idiopathic (PE) occurred, a rare finding in Pheo, contrasting with reports of deep venous thrombosis or vasculitis.^6,7^ While a pro-thrombotic state is recognized in malignant Pheo, the aetiology in benign Pheo remains unclear.^8,9^ Although rare, thrombotic complications such as PE have been described in association with Pheo. Several mechanisms may contribute to this hypercoagulable state: catecholamine excess promotes vasoconstriction, endothelial injury, and platelet aggregation, all of which increase thrombosis risk. Elevated circulating catecholamines may also lead to haemoconcentration and reduced fibrinolysis. In our case, no provoking factors for PE were identified, supporting the hypothesis of a Pheo-induced pro-thrombotic state. This is further supported by previous reports linking Pheo to idiopathic venous thromboembolism, although the pathophysiological basis remains incompletely understood.^10^

The occurrence of PE in our patient may also have contributed to the adrenergic stimulation and acted as a precipitating factor for ITC, suggesting a complex interplay between catecholamine excess, myocardial dysfunction, and thrombosis.^11^ Catecholamines may increase platelet aggregation and thrombosis risk. Pulmonary embolism can trigger or result from TTC, though TTC-associated idiopathic PE is rare.^10,11^

To our knowledge, this is the third reported case of PE in Pheo with ITC, one diagnosed post-mortem.^12,13^ Of the two previous published cases, one was diagnosed post-mortem, and the other reported only limited haemodynamic monitoring data, with no detailed description of therapeutic strategies or timing of medication adjustments.^12,13^ In contrast, our case provides continuous intra-arterial BP and HR monitoring that revealed cyclical haemodynamic fluctuations with a reciprocal pattern, an uncommon but diagnostically significant feature in Pheo. Moreover, our patient experienced paroxysmal hypertensive crises alternating with severe hypotension, a rare and challenging presentation in the context of suspected ITC and PE. Furthermore, this case underscores the importance of maintaining a high index of suspicion for Pheo in patients with unexplained cyclic BP/HR variability, ITC, and idiopathic PE. The identification of the adrenal mass was incidental during imaging for PE, but the distinctive haemodynamic pattern and recurrence of adrenergic symptoms pointed towards catecholamine excess as a unifying diagnosis. Additionally, our report illustrates the rationale for transitioning to α-blockade (urapidil followed by doxazosin) and cautious β-blockade only after stabilization, a strategy aligned with best practices but rarely documented in acute settings with such detailed temporal data. The triad BP variability + HR variability + chest pain (Mazza–Zuin triad) likely depends on which Pheo phenotype. Noradrenergic phenotypes have higher atherosclerotic complications, while adrenergic phenotypes, as in this case, are linked to acute myocardial damage resembling TTC.^14,15^

## Conclusions

In conclusion, BP fluctuations with alternating hypotension and hypertension during ITC should suggest hidden Pheo. Coexisting PE complicates clinical management, alters haemodynamics, and raises bleeding risks. A collaborative approach involving cardiologists, hypertension specialists, and surgeons is mandatory.

## Lead author biography


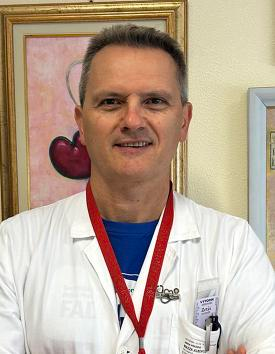


Dr Alberto Mazza is the head of the European Hypertension Excellence Centre at the Rovigo Hospital. A graduate in medicine and surgery from the University of Padua, he specialized in internal medicine and clinical nutrition and obtained a PhD in diabetology, nephro-urology, medical therapy, and clinical pharmacology. Since 2006, he has been a medical director at the Rovigo Hospital, within the UOC (Complex Operative Unit) of Internal Medicine of the ULSS 5 Polesana Health Authority. In 2008, he specialized in hypertension at the European Society of Hypertension. He is currently the director of the internal medicine department of Adria and its ESH (European Society of Hypertension) Excellence Centre for arterial hypertension.

## Supplementary Material

ytaf574_Supplementary_Data

## Data Availability

The data underlying this article could be shared upon reasonable request to the corresponding author.

## Supplementary material

**Consent:** Written informed consent was obtained from the patient for the publication of this case report and the accompanying images in line with the COPE guidelines.

**Funding:** None declared.
