# Supplementary material for: A case report of blood pressure fluctuation, inverted Takotsubo cardiomyopathy, and pulmonary embolism: searching for a link
Source: Eur Heart J Case Rep. 2025 Nov 1;9(11):ytaf574. doi: 10.1093/ehjcr/ytaf574 (PMC12626123; doi:10.1093/ehjcr/ytaf574)
Supplement: ytaf574_Supplementary_Data [file ytaf574_supplementary_data.docx]

**A Case Report of Blood pressure fluctuation, inverted Takotsubo cardiomyopathy and pulmonary embolism: searching for a link.**

Supplementary Files


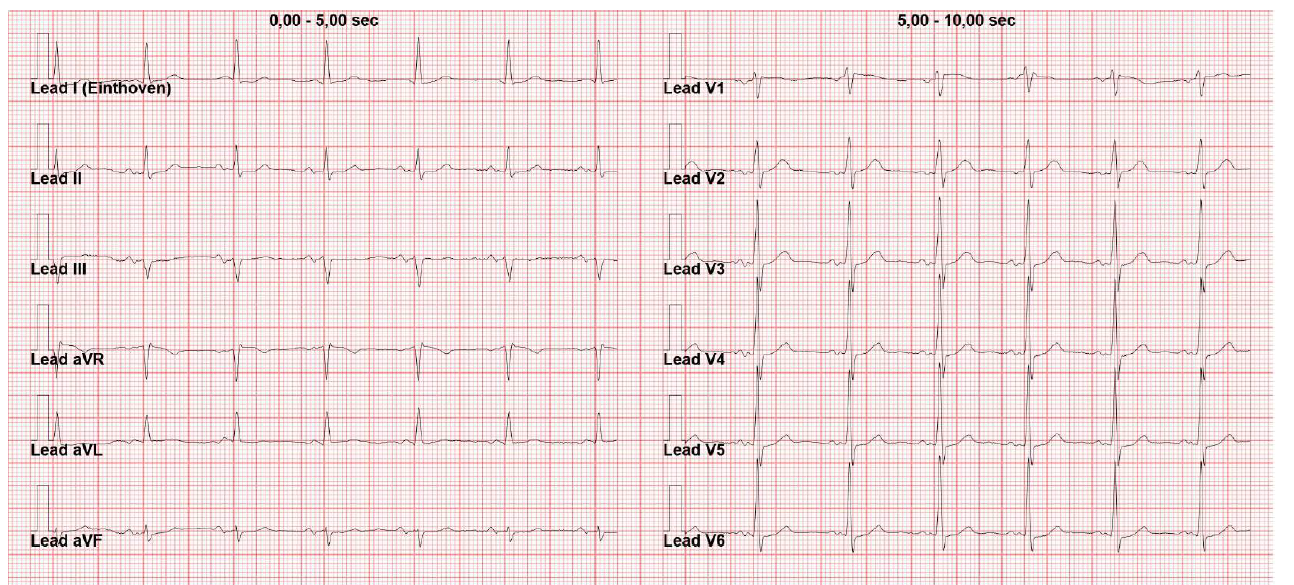


**Supplementary Figure 1.** Twelve-lead electrocardiogram showing sinus rhythm with incomplete right bundle branch block, left axis deviation, interventricular conduction delay, and nonspecific ST-T changes in the right precordial leads.
